# Visual and Non-Visual Contributions to the Perception of Object Motion during Self-Motion

**DOI:** 10.1371/journal.pone.0055446

**Published:** 2013-02-07

**Authors:** Brett R. Fajen, Jonathan S. Matthis

**Affiliations:** Department of Cognitive Science, Rensselaer Polytechnic Institute, Troy, New York, United States of America; Bielefeld University, Germany

## Abstract

Many locomotor tasks involve interactions with moving objects. When observer (i.e., self-)motion is accompanied by object motion, the optic flow field includes a component due to self-motion and a component due to object motion. For moving observers to perceive the movement of other objects relative to the stationary environment, the visual system could recover the object-motion component – that is, it could factor out the influence of self-motion. In principle, this could be achieved using visual self-motion information, non-visual self-motion information, or a combination of both. In this study, we report evidence that visual information about the speed (Experiment 1) and direction (Experiment 2) of self-motion plays a role in recovering the object-motion component even when non-visual self-motion information is also available. However, the magnitude of the effect was less than one would expect if subjects relied entirely on visual self-motion information. Taken together with previous studies, we conclude that when self-motion is real and actively generated, both visual and non-visual self-motion information contribute to the perception of object motion. We also consider the possible role of this process in visually guided interception and avoidance of moving objects.

## Introduction

When humans and other animals move through the world, their own movement is often accompanied by the movement of other objects. Depending on the situation, these other objects could be targets to be intercepted, obstacles to be avoided, or merely objects whose movement is of interest. For many species, the ability to perceive object motion during self-motion and to coordinate one’s own movement with the movement of other objects is achieved by relying on information in optic flow – that is, the streaming pattern of optical motion generated by movement through the environment [1].

**Figure 1:**
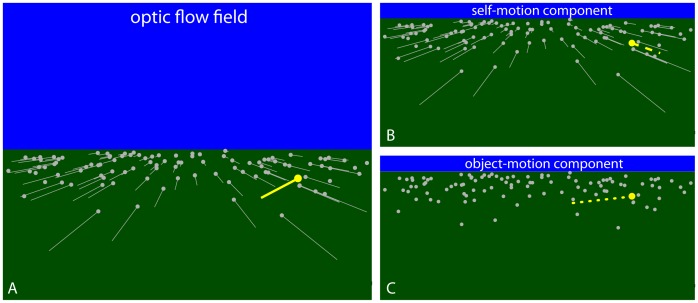
Optic flow field and decomposition into self-motion and object-motion components. (A) Optic flow field generated by an observer moving over a ground surface and an object (yellow dot) moving from right to left. (B) The component of optic flow due to self-motion independent of object motion. (C) The component of optic flow due to object motion independent of self-motion. The optic flow field (A) is the vector sum of the self-motion (B) and object-motion (C) components.

When self-motion is accompanied by object motion, the resultant optic flow includes a self-motion component caused by the observer’s movement through the world and an object-motion component caused by the movement of objects; that is, the optic flow field (Figure 1A) is the vector sum of the self-motion (Figure 1B) and object-motion components (Figure 1C). The optical motion of the stationary background (represented by the gray vectors in Figure 1A) contains only the self-motion component and is characterized by a radially expanding pattern with a focus of expansion aligned with the direction of self-motion [1], [2]. The optical motion of the moving object (represented by the yellow vector in Figure 1A) includes both the self-motion and object-motion components. Unless the object is moving parallel to the observer, the direction of optical motion deviates from the radially expanding background flow, allowing for the detection of moving objects during self-motion [3].

Visual information that moving observers could use to perceive object motion relative to the stationary environment (i.e., in world coordinates) is found in the object-motion component of optic flow. This means that for moving observers to perceive object motion in world coordinates, the visual system should be capable of recovering the object-motion component of optic flow. Because the optic flow field is the sum of the self-motion and object-motion components, the object-motion component can be recovered by factoring out the influence of the self-motion component [4]. The component that remains reflects the optic flow due to object motion independent of the optic flow due to the observer (Figure 1C).

In principle, factoring out the self-motion component of optic flow could be achieved using visual information about self-motion, non-visual information about self-motion (e.g., from proprioception, inertial cues, motor efference), or some combination of both. The contribution of visual self-motion information was recently investigated using computer-generated stimuli simulating combined self-motion and object motion. When subjects viewed these stimuli on a monitor [5], [6] or in a CAVE [7], the perceived trajectory of the moving object was shifted as if the visual system was using the optic flow from the stationary background to factor out the influence of self-motion. The influence of self-motion on object motion persisted with only a small decrease in strength when the moving object and background optic flow were in different parts of the visual field [6], [7], ruling out an account based on local motion contrast. Instead, it was concluded that the component of optic flow due to object motion is recovered through a process called *flow parsing* [6], [8], [9], in which visual self-motion information is used to identify and globally discount the component of optic flow due to self-motion.

The aforementioned studies demonstrate a role for visual self-motion information. However, subjects in those studies were not actually moving. Self-motion was simulated and viewed on a monitor or projection screen by a stationary observer. As such, none of the sources of non-visual self-motion information that normally accompany locomotion and are known to contribute to the perception of self-motion [10], [11], [12] were consistent with actual self-motion. Thus, the contribution of visual self-motion information when reliable non-visual self-motion information is also available remains an open question.

One possibility is that visual self-motion information dominates; that is, non-visual self-motion information does not contribute even during actively generated self-motion. In earlier studies, however, it has been shown that non-visual self-motion information does influence the perception of object motion while walking [13] and when making head movements [14] even when visual self-motion information is also available. Non-visual self-motion information has also been implicated in the perception of a stable environment during self-motion [4]. These previous studies rule out the possibility that visual self-motion information completely dominates non-visual self-motion information, at least when both are available and are comparable in terms of reliability.

In the present study, we consider two other hypotheses: (1) non-visual self-motion information dominates visual self-motion information such that visual information does not play a role when self-motion is real, and (2) both visual and non-visual self-motion information contribute. To test these hypotheses, we conducted two experiments in an ambulatory virtual environment that was viewed through a head-mounted display (HMD). In both experiments, subjects performed an affordance perception task that involved making judgments about how to avoid moving obstacles. The task provided an ecologically valid context within which to study the contribution of visual and non-visual self-motion information.

### Experiment 1

**Figure 2:**
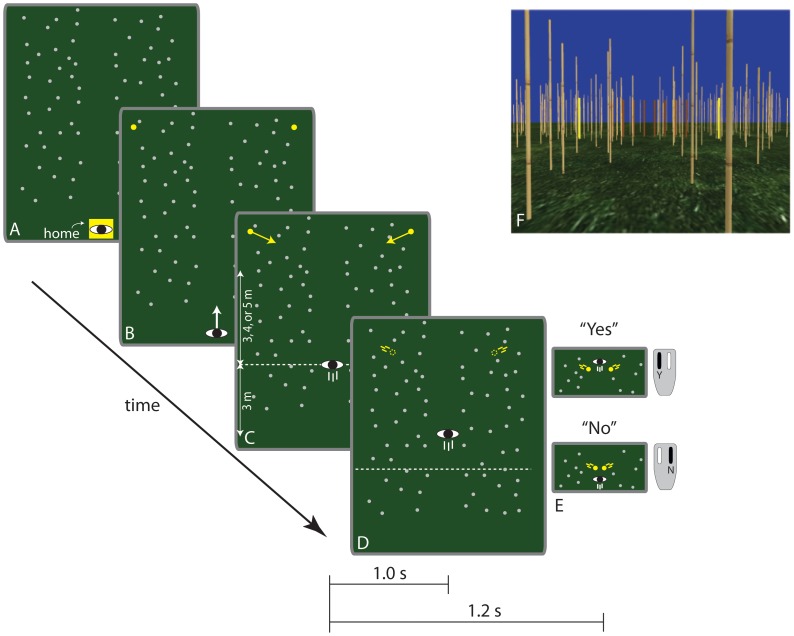
Sequence of events in Experiments 1. (A–E) Sequence of events on each trial in Experiment 1. (F) Screenshot of virtual environment viewed through the HMD.

Figure 2 illustrates the task that subjects performed in Experiment 1. Subjects began each trial by walking to a designated home location and turning until they were facing a path lined with bamboo-textured posts (Figure 2A). When they pressed a button on a handheld remote mouse, two stationary yellow cylinders appeared at the same depth and positioned symmetrically about the path (Figure 2B). The appearance of the cylinders cued subjects to begin walking down the path. When subjects walked 3 m from the home location, the cylinders began to converge to a point along the path (Figure 2C). The cylinders moved for 1 s before they disappeared (Figure 2D). The subjects’ task was to press one of two buttons on the remote mouse within 1.2 s of cylinder movement to indicate whether they could safely pass through the gap before it closed without running and without rotating their shoulders (Figure 2E). In other words, subjects were instructed to base their judgment on whether they could have passed through the gap if they had been allowed to walk as quickly as possible. Note that this is not the same as judging whether one would have passed through the gap if one’s current locomotor speed is maintained. The task used in this experiment required subjects to perceive how fast they needed to move to pass in front of the obstacle in relation to how fast they were capable of moving, rather than in relation to how fast they were currently moving. Subjects were not given any specific instructions regarding where to look. Figure 2F shows a screenshot of the virtual environment viewed through the HMD.

**Figure 3:**
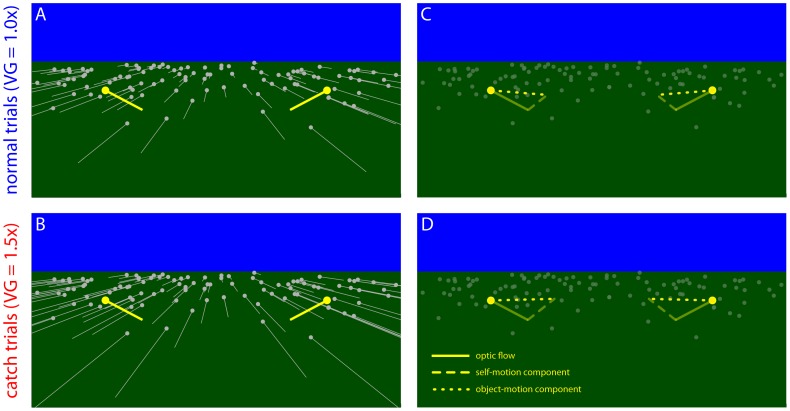
Experiment 1 predictions. Predictions for normal and catch trials in Experiment 1. (A) and (B) depict the optic flow field on normal trials and catch trials, respectively. Gray vectors depict the optic flow of the stationary background and yellow vectors depict the optic flow of the moving objects. (C) and (D) show the object-motion component (dotted lines) and how it is recovered by subtracting the self-motion component (faded dash lines) from the optic flow of the moving objects.

The distance that subjects would have to walk to pass through the gap and the time-to-closure of the gap were manipulated across trials yielding a range of conditions that varied from passable even at a slow walking speed to impassable even at a fast walking speed. There were three distances, five time-to-closures, and eight repetitions per condition, yielding 120 trials. In addition, there were also 24 randomly interspersed catch trials, which differed from normal trials in that the speed with which subjects moved through the virtual environment relative to the real world (i.e., the *visual gain*) was increased by 50%. The difference in visual gain is illustrated in Figures 3A and 3B by the difference in the lengths of the gray vectors, which depict the optic flow from the ground plane. The ground flow vectors are longer in Figure 3B (catch trials) than in Figure 3A (normal trials) due to the difference in visual gain.

Importantly, the visual gain manipulation affected subjects’ motion relative to stationary features of the environment (i.e., the ground surface, the bamboo posts, and the cylinders before they began moving) but did not affect their movement relative to the cylinders after the onset of cylinder motion. That is, visual gain with respect to the moving cylinders was identical on normal trials and catch trials. This is illustrated in Figures 3A and 3B. Although the gray vectors corresponding to the ground flow are longer in Figure 3B (catch trials), the yellow vectors corresponding to the moving object are the same in both figures. Thus, the only difference between normal trials and catch trials was that visual information about self-motion provided by optic flow from the stationary elements of the scene (e.g., the textured ground plane, the bamboo-textured posts, and the cylinders before they began moving) was manipulated on catch trials. This means that any differences in subjects’ responses can be attributed to the effect of the manipulation of visual self-motion information, and not to differences in the local optical motion of the objects.

The way in which the manipulation of visual gain affects judgments depends on the contribution of visual self-motion information to the recovery of the object-motion component of optic flow. If subjects rely on visual self-motion information, as suggested by Warren and Rushton [6], [8], the component that is attributed to self-motion (i.e., the component that is factored out) should be greater on catch trials (compare faded dashed lines in Figure 3C and 3D). Because the remaining component points farther down the locomotor axis, the obstacles should be perceived as converging toward a point that is farther away. Therefore, the likelihood that subjects perceive the gap as passable should decrease on catch trials compared to normal trials with the same initial conditions. On the other hand, if people rely entirely on non-visual self-motion information, then because the manipulation of visual gain does not affect non-visual self-motion information, judgments on normal trials and catch trials should be similar. Thus, the visual hypothesis predicts a decrease in the percentage of passable judgments and the non-visual hypothesis predicts no change.

### Methods

#### Ethics statement

The experimental protocol was approved by the Institutional Review Board at Rensselaer Polytechnic Institute and is in compliance with the Declaration of Helsinki. All subjects gave informed consent in writing before participating in the experiment.

#### Participants

Fourteen subjects (4 female, 10 male, mean age: 22.5 years) participated in Experiment 1. Subjects were compensated for participation with either extra credit or payment at $12/hr. Data from one subject were excluded because there were too few “yes” responses to permit analysis. Data from another subject were excluded because responses did not vary systematically with required speed suggesting that the subject either did not understand the task or ignored the instructions.

#### Equipment

The experiment was conducted in a 6.5 m ×9 m ambulatory virtual environment laboratory equipped with a nVis nVisor SX HMD which generated stereo images at 60 Hz. The HMD weighs 1000 g, the resolution is 1280 pixels ×1024 pixels per eye, and the diagonal field-of-view is 60°. Head position and orientation were tracked using an Intersense IS-900 hybrid sonic/inertial system. Data from the head tracker were used to update the position and orientation of the simulated viewpoint. The cables from the HMD and tracking system were bundled together and held by the experimenter, who walked alongside the participant as he or she moved to ensure that the cables did not interfere with the subject’s movement. The virtual environment was created using Sense 8 World Tool Kit software running on a Dell Workstation 650 with a Wildcat 7110 graphics card.

#### Virtual environment and procedure

The virtual environment consisted of a grass textured ground plane, a solid blue sky, and an array of randomly distributed bamboo-textured posts that lined a walkway extending out in front of the starting location (see Figure 2F). The moving obstacles were 2.0 m tall solid yellow cylinders with a radius of 0.05 m. Their initial positions varied randomly (±0.5 m) on each trial around a default initial position 5.5 m in depth and 2.0 m to the side. The cylinders were always positioned symmetrically about the midline, as illustrated in Figure 2. The trajectories of the obstacles were manipulated by varying the distance and time-to-closure of the gap. Distance was defined as the distance from the position of the subject at the moment that the obstacles began moving to the position of the cylinders when the gap between the cylinders was equal to the subject’s shoulder width. The time-to-closure of the gap was defined as the amount of time it took for the size of the gap to reach one shoulder width, which was measured for each subject prior to the beginning of the experiment and used by the program to determine the trajectories of the moving obstacles. Together, distance and time-to-closure determined the direction and speed of the obstacle. Thus, the obstacle approached the observer more quickly when distance was shorter and moved toward the locomotor axis more quickly when time-to-closure was shorter. For normal trials, there were three distances (3, 4, and 5 m), five time-to-closures (1.6, 1.8, 2.0, 2.2, and 2.4 s), and 8 repetitions per condition. For catch trials, there were three distances (3, 4, and 5 m) and two time-to-closures (1.8 and 2.0 s). The 24 catch trials were randomly intermixed with the 120 normal trials yielding 144 trials per session. The decision to present a high ratio of trials with normal visual gain to trials with faster-than-normal visual gain was necessary to avoid adaptation effects. In a previous study [13], we found that when subjects perform the aforementioned task with visual gain set to 1.5× on the majority of trials, they adapted to the faster-than-normal visual gain, which affects the perceived motion of the obstacles. Had we manipulated visual gain as an independent variable with an equal number of trials with normal and faster-than-normal visual gains, adaptation effects may have contaminated the results. Therefore, we chose to manipulate visual gain by adding a small number of catch trials.

Prior to the start of each session, subjects completed a 24-trial warm up task designed to familiarize them with moving in the virtual environment. The warm up task required subjects to walk to catch virtual fly balls that were projected into the air. Subjects caught fly balls using their right hand, the position and orientation of which was tracked using an Intersense IS-900 hand tracker. Subjects also completed 8 trials of the experimental task before the experiment began in order to ensure that they understood the instructions.

#### Data Analysis

The dependent measure was the percentage of yes responses on normal trials and catch trials. To ensure that visual gain was the only factor that differed across trial type, we compared responses on catch trials with responses on the subset of normal trials with initial conditions that matched the initial conditions on catch trials (i.e., 3 m/1.8 s, 3 m/2.0 s, 4 m/1.8 s, 4 m/2.0 s, 5 m/1.8 s, and 5 m/2.0 s). The data from these conditions were collapsed across distance and time-to-closure and the overall percentage of yes responses was calculated.

**Figure 4:**
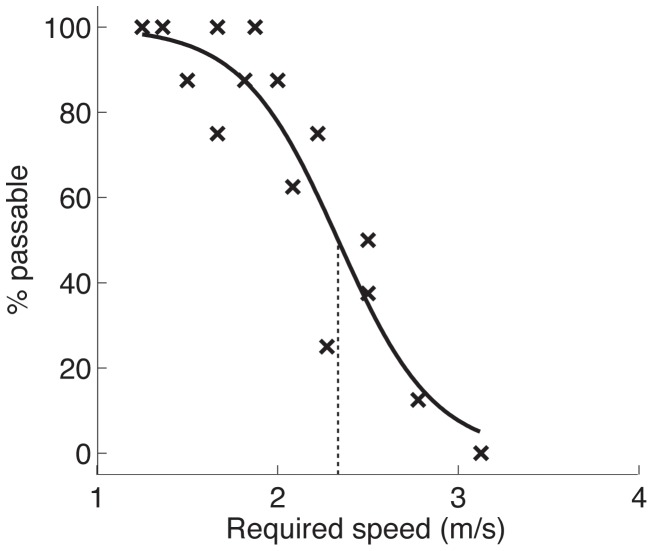
Sigmoid fit. Percentage of passable judgments as a function of required speed for a representative subject. Solid black curve represents best-fitting sigmoid function and dotted line indicates critical value of required speed.

In addition, we used the percentage of yes responses to estimate the critical value of required speed below which subjects tended to perceive the gap as passable and above which they tended to perceive the gap as impassable. The first step in the critical value analysis was to calculate the minimum locomotor speed required to safely pass through the gap for each combination of distance and time-to-closure. Note that minimum locomotor speed was unaffected by the manipulation of visual gain. That is, for pairs of conditions with the same distance and time-to-closure, minimum locomotor speed was the same on normal trials and catch trials. This is because the manipulation of visual gain affected subjects’ movement relative to the stationary background but not their movement relative to the moving objects. Therefore, the speed that subjects had to walk (in real world coordinates) to safely pass through the gap was not affected by the visual gain manipulation. The next step was to plot the percentage of passable responses as a function of minimum required speed for each individual subject. Subjects tended to judge trials with slow required speeds as passable and trials with faster required speeds as impassable. The data from each individual subject were fit with a sigmoid function and the point at which the best fitting curve crossed 50% was interpreted as the critical value of required speed below which the subject tended to make passable responses and above which the subject tended to make impassable responses (see Figure 4). Critical required speed was calculated for both normal trials and catch trials for each subject and then averaged across subjects to arrive at a mean critical required speed for both conditions.

The predictions for critical required speed are the same as those for the percentage of passable judgments. That is, if subjects rely on visual self-motion information, then critical required speed should be lower on catch trials (just as the percentage of passable judgments should be lower on catch trials). Likewise, if subjects rely entirely on non-visual self-motion information, then critical required speed should be the same on catch trials and normal trials. The point of including the analysis of critical required speed is that it also allows us to measure the magnitude of the effect. As shown in the results section below, we can compare the magnitude of the effect to that which would be expected if subjects relied entirely on visual self-motion information.

In this particular study, the accuracy of judgments was less of a concern than the effects of manipulations of self-motion information. Therefore, the experiment was not designed to measure the accuracy with which subjects judged whether the gap was passable. However, we did measure accuracy in an earlier experiment reported in [15]. In Experiment 3 of that study, subjects judged whether they could have safely passed through the gap on some trials and actually attempted to pass through the gap on other trials. Judgments were closely matched to actions with no systematic bias to overestimate or underestimate passability.

## Results

Before considering the effect of the visual gain manipulation, it was necessary to confirm that judgments were affected by time-to-closure and distance. For this analysis, we focused on normal trials due to the wider range of time-to-closures and distances that were used on such trials. Table 1 shows the mean percentage of passable judgments as a function of time-to-closure and distance. As expected, subjects tended to perceive gaps as passable when time-to-closure was long and distance was short (and vice-versa). The main effects of both time-to-closure (F_4, 44_ = 65.48, p<.01, partial η^2^ = .856) and distance (F_2, 22_ = 194.17, p<.01, partial η^2^ = .946) were significant as was the interaction (F_8, 88_ = 3.86, p<.01, partial η^2^ = .26).

**Table 1:** Percentage of passable judgments in Experiment 1.

Time-to-closure (s)	Trial type	Distance (m)						
		3		4		5		
		M	SE	M	SE	M	SE	
1.6	Normal	59.4	8.3	17.7	5.4	10.4	4.3	
	Catch	–	–	–	–	–	–	
1.8	Normal	82.3	5.6	38.5	7.9	11.5	3.6	
	Catch	66.7	7.7	39.6	8.9	6.3	3.3	
2.0	Normal	87.5	4.6	61.5	7.8	26.0	7.6	
	Catch	79.2	7.4	35.4	6.5	14.6	4.8	
2.2	Normal	88.5	3.2	71.9	6.6	25.0	5.5	
	Catch	–	–	–	–	–	–	
2.4	Normal	100.0	0.0	86.5	3.6	53.1	8.0	
	Catch	–	–	–	–	–	–	

Before any differences in responses on normal trials and catch trials can be attributed to the manipulation of visual gain, it is necessary to confirm that walking behavior was the same on both trial types. We calculated mean head speed on both normal trials and catch trials between the moment that the obstacles began to move and the moment that a response was recorded. The mean difference in head speed on normal trials and catch trials was nearly zero (*M* = 0.03 m/s). We also calculated the mean lateral position of the head at the moment that the response was recorded and again found that the difference was very small (*M* = 0.02 m). Therefore, walking speed and direction were nearly identical on normal trials and catch trials, ruling out the possibility that any differences in responses across trial types could be due to differences in walking behavior.

**Figure 5:**
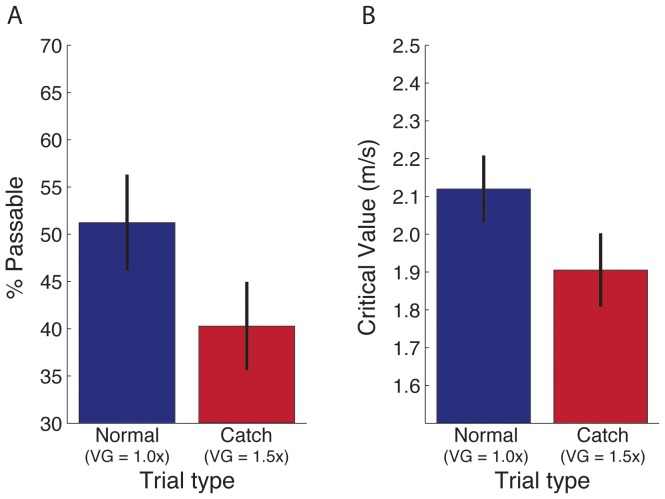
Experiment 1 results. Percentage of passable judgments (A) and critical value of required speed (B) in Experiment 1. Error bars indicate ±1 SE. Note that the estimate of critical required speed on normal trials in (B) is based on data from all 15 initial conditions. It is also possible to estimate critical required speed based on the data from the subset of normal trials with initial conditions that match those on catch trials. When critical required speed is estimated using the latter method, the mean and standard error (M = 2.14 m/s, SE = 0.09) are nearly identical to the mean and standard error based on the data from all normal trials (M = 2.12 m/s, SE = 0.09).

Figure 5A shows the mean percentage of “passable” judgments on normal and catch trials. Recall that the percentage of passable judgments on normal trials includes only those trials with initial conditions that matched the initial conditions on catch trials. This was necessary to ensure that visual gain was the only factor that differed across trial type. Consistent with an influence of visual self-motion information, subjects were significantly less likely to perceive gaps as passable on catch trials (t_11_ = 4.62, p<.01). This effect was consistent across conditions, as shown in Table 1.

As predicted by the visual hypothesis, critical required speed was faster on normal trials compared to catch trials (t_11_ = 3.57, p<.01; see Figure 5B). However, if subjects relied entirely on visual self-motion information, the critical value of required speed should decrease on catch trials by 50% of the speed at which the subject walked. Using the data from the head tracker to estimate walking speed, we calculated for each subject the decrease in critical required speed that was predicted by the visual hypothesis. The mean ratio of the actual decrease to predicted decrease was 0.28; that is, the magnitude of the effect of visual gain was only 28% of magnitude that would be expected if subjects relied entirely on visual self-motion information.

The difference between the magnitude predicted by the visual hypothesis and the magnitude that was observed is not surprising in light of previous findings indicating that the perception of object motion is influenced by both visual and non-visual self-motion information. Recall that when self-motion is real and actively generated, non-visual self-motion information is also known to play a role in recovering the object-motion component of optic flow [13]. If subjects in the present experiment relied on both visual self-motion information (which was manipulated) and non-visual self-motion information (which was not manipulated), then one would expect the manipulation of visual gain to influence judgments but not as much as would be expected if subjects relied on visual self-motion information alone. Therefore, the findings are consistent with the use of both visual and non-visual self-motion information when self-motion is real and actively generated.

The contribution of non-visual self-motion information explains why the effect of visual self-motion information was less than 100% (specifically, 28%). However, it does not necessarily follow that the remaining 72% was due to the influence of non-visual self-motion information. There are two additional factors that also could have influenced judgments. First, our estimate of the strength of the visual gain manipulation was based on a theoretical prediction that assumes that the visual system completely compensated for the faster-than-normal optic flow on catch trials. That is, it assumes that the component that was attributed to self-motion was greater on catch trials by an amount that corresponds to the increase in visual gain (i.e., 50%). Given the large increase in visual gain, it is possible that the visual system only partially compensated [16] such that the component of optic flow that was attributed to self-motion was less than 50% greater on catch trials compared to normal trials. If so, then the magnitude of the expected effect would be less than if the visual system completely compensated. Therefore, the contribution of visual self-motion information relative to non-visual self-motion information may be more than is suggested by the estimate of 28%.

Second, the increase in visual gain may have led subjects to feel that their speed of self-motion increased and they were suddenly capable of moving faster, which could make them more likely to perceive gaps as passable. Such an effect is plausible, given that global optic flow rate is known to influence perceived speed of self-motion [17]. If, in fact, increasing visual gain caused subjects to perceive that their locomotor capabilities were suddenly enhanced, the visual gain manipulation may have actually led to an increase in the likelihood of perceiving gaps as passable. Thus, even if the visual gain manipulation caused subjects to perceive that the obstacles were converging toward a point that was farther away, the effect on judgments may have been partially cancelled if subjects also perceived that they could move faster.

In an earlier study using a similar task and design [13], we isolated the effect of increasing visual gain on perceived locomotor capabilities and found that the effect was weak, resulting in a mere 7% increase in critical required speed. This means that if it was possible to eliminate the influence of visual gain on felt locomotor capabilities, then the critical required speed on catch trials might have decreased a bit more than depicted in Figure 5B. That is, change in critical required speed on catch trials might have been slightly greater. This is another reason why the relative contribution of visual self-motion information may be greater than is suggested by the estimate of 28%.

To summarize, the findings of Experiment 1 demonstrate that visual self-motion information influences the perception of object motion even when self-motion is real and actively generated. However, the strength of the effect was less than would be expected if subjects relied entirely on visual self-motion information. We attribute this to the influence of non-visual self-motion information, which was demonstrated in Fajen and Matthis [13]. Thus, the findings of Experiment 1 together with the effects reported in Fajen and Matthis [13] support the idea that both classes of information contribute to the perception of object motion.

### Experiment 2

**Figure 6:**
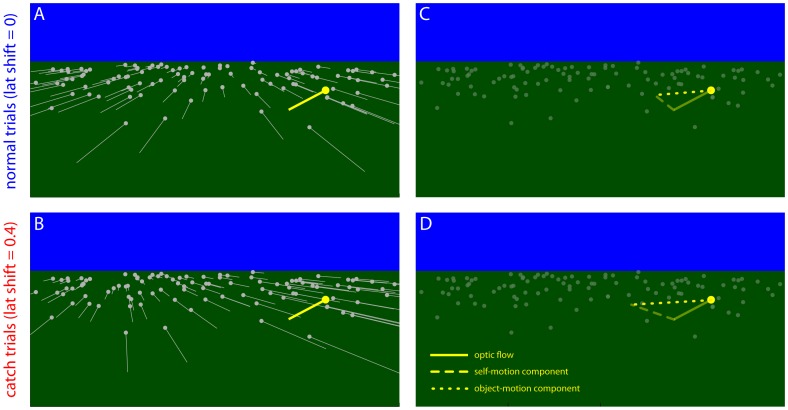
Experiment 2 predictions. Predictions for normal and catch trials in Experiment 2 for a trial in which the object moves from right to left. (A) and (B) depict the optic flow field on normal trials and catch trials, respectively. Gray vectors depict the optic flow of the stationary background and yellow vectors depict the optic flow of the moving objects. (C) and (D) show the object-motion component (dotted lines) and how it is recovered by subtracting the self-motion component (faded dash lines) from the optic flow of the moving objects.

Whereas Experiment 1 focused on the role of visual information about the speed of self-motion, Experiment 2 was designed to investigate the role of visual information about the direction of self-motion. The task and experimental design were similar with the following exceptions. First, there was a single obstacle (rather than two) that appeared to the right or left of the path and moved inward. Subjects were instructed to judge whether they would have passed in front of or behind the obstacle. Second, the direction rather than the speed of locomotion was manipulated on catch trials (see Figure 6A–B). As subjects walked, their heading in the virtual environment was shifted to the left (on trials in which the object moved from right to left) or to the right (on trials in which the object moved from left to right). This was achieved by laterally shifting subjects’ position in the virtual environment to the left or right relative to the stationary features of the virtual environment by an amount that was proportional to their displacement along the locomotor axis. The proportionality constant was 0.4. Therefore, if the subject’s displacement along the locomotor axis between successive frames was Δz, then his or her position in the virtual environment was laterally shifted by 0.4× Δz, which corresponds to a shift of ∼22°. As in Experiment 1, the manipulation on catch trials affected subjects’ movement relative to the stationary features of the environment but not relative to the moving cylinder. Thus, any differences in judgments between normal and catch trials can be attributed to the manipulation of visual information about the direction of self-motion.

If people rely on visual information about the direction of self-motion to recover the object-motion component of optic flow, the component of optic flow that is attributed to self-motion (i.e., the component that is factored out) should be greater on catch trials (compare dashed lines in Figures 6C and 6D). Therefore, the component that remains points farther to the left (for trials in which the object is moving leftward as in Figure 6), which means that subjects should perceive that the obstacle is moving leftward at a faster rate and be less likely to perceive that they can pass in front of the obstacle on catch trials. On the other hand, if subjects rely entirely on non-visual self-motion information, responses on catch trials should not be affected by the lateral shift.

### Methods

#### Participants

Fourteen subjects (6 female, 8 male, mean age: 22.1 years), none of whom were in Experiment 1, participated in Experiment 2. As in Experiment 1, subjects were compensated for participation with either extra credit or payment at $12/hr. Data from one subject were excluded because there were too many “yes” responses to calculate a critical value of required speed. Data from another subject were excluded because responses did not vary systematically with required speed suggesting that the subject either did not understand the task or ignored the instructions.

#### Virtual environment and procedure

The virtual environment and procedure used in Experiment 2 were similar to Experiment 1 with a few exceptions, two of which were noted above (i.e., one obstacle rather than two, manipulation of lateral shift rather than visual gain). In addition, recall that subjects in Experiment 1 walked for 3 m before the cylinders began moving. Because the lateral shift manipulation perturbed subjects’ direction in the virtual environment, it was necessary to eliminate the 3 m approach phase in Experiment 2. Had there been an approach phase in Experiment 2, it would have been difficult to ensure that subjects were in the same position on normal trials and catch trials at the moment that the cylinder began moving. Therefore, the obstacle appeared and began moving at the same time that the auditory signal to begin walking was presented.

As in Experiment 1, the trajectory of the moving obstacle was manipulated by varying the distance and time-to-closure. With a single obstacle, the distance corresponds to the distance along the locomotor axis from the position of the subject at the beginning of the trial to the position of the obstacle when its distance to the locomotor axis was equal to one-half of the subject’s shoulder width. The time-to-closure was the amount of time it took for the obstacle to reach this point. For normal trials, there were three distances (3, 4, and 5 m), five time-to-closures (2.2, 2.4, 2.6, 2.8, and 3.0 s), and 8 repetitions per condition. For catch trials, the following six distance/time-to-closure pairings were: 3 m/3.0 s, 3 m/2.6 s, 4 m/3.0 s, 4 m/2.8 s, 4 m/2.4 s, and 5 m/2.8 s. These particular conditions were chosen for catch trials because pilot testing revealed that subjects consistently judged the obstacle as passable at the slowest required speed (3 m/3 s) and impassable at the highest required speed (5 m/2.8 s). Each pairing was repeated four times, yielding 24 catch trials that were randomly intermixed with the 120 normal trials. The longer time-to-closure values were used because subjects were stationary rather than walking as in Experiment 1 when the obstacle first appeared and began moving.

### Results

As in Experiment 1, we first confirmed that judgments on normal trials were affected by time-to-closure and distance. As shown in Table 2, subjects tended to perceive gaps as passable when time-to-closure was long and distance was short (and vice-versa), yielding significant main effects of both time-to-closure (F_4, 44_ = 59.50, p<.01, partial η^2^ = .84) and distance (F_2, 22_ = 143.33, p<.01, partial η^2^ = .93). The interaction was also significant (F_8, 88_ = 3.49, p<.01, partial η^2^ = .24).

**Table 2:** Percentage of passable judgments in Experiment 2.

Time-to-closure (s)	Trial type	Distance (m)					
		3		4		5	
		M	SE	M	SE	M	SE
2.2	Normal	65.6	8.0	19.8	4.2	4.2	3.2
	Catch	–	–	–	–	–	–
2.4	Normal	85.4	4.0	32.3	7.3	7.3	2.9
	Catch	–	–	12.5	4.9	–	–
2.6	Normal	84.4	4.6	50.0	8.6	26.0	7.9
	Catch	62.5	9.5	–	–	–	–
2.8	Normal	88.5	4.2	67.7	6.0	19.8	4.7
	Catch	–	–	18.8	7.6	16.7	7.1
3.0	Normal	93.8	2.4	77.1	5.9	39.6	7.8
	Catch	79.2	6.8	45.8	6.8	–	–

As in Experiment 1, walking behavior was the same on normal trials and catch trials. The mean difference in head speed between the two trials types in Experiment 2 was just 0.01 m/s, and the mean difference in the lateral position of the head was just 0.01 m/s. Thus, we can again rule out the possibility that any differences in responses across trial types were due to differences in walking behavior.

**Figure 7:**
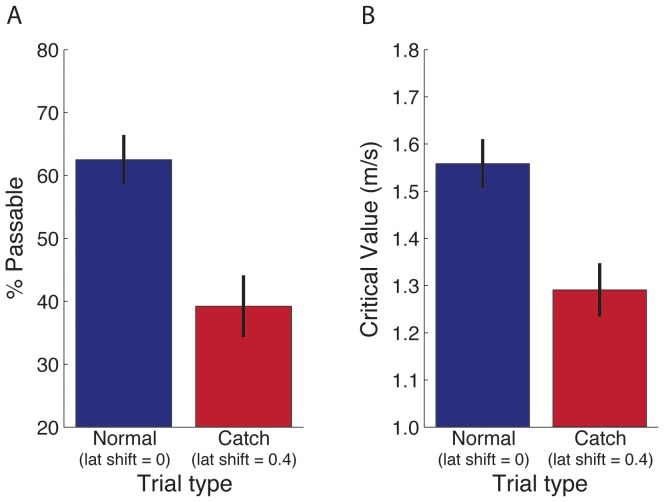
Experiment 2 results. Percentage of passable judgments (A) and critical value of required speed (B) in Experiment 2. Error bars indicate ±1 SE. As in Experiment 1, critical required speed can also be estimated using the subset of normal trials with initial conditions that match those on catch trials. Using this method, the critical required speed (M = 1.54 m/s, SE = 0.05) is nearly identical to the critical required speed based on all initial conditions (M = 1.56 m/s, SE = 0.05).

As predicted by the visual hypothesis, subjects were less likely to perceive that they could pass in front of the obstacle on catch trials (t_11_ = 4.90, p<.01; see Figure 7A). Again, this effect was consistent across conditions, as shown in Table 2. The critical value of required speed was significantly lower on catch trials (t_11_, = 4.93, p<.01; see Figure 7B). However, the magnitude of the effect of lateral shift was only 26% of that which would be expected if subjects relied entirely on visual self-motion information.

The results of Experiment 2 as well as our interpretation mirror those of Experiment 1. The fact that the magnitude of the effect was less than 100% can be attributed to an influence of non-visual information about the direction of self-motion. As in Experiment 1, we cannot draw any strong quantitative conclusions about the precise relative contributions of visual and non-visual information about the direction of self-motion. However, the results of Experiment 2 suggest that when self-motion is real and actively generated, both visual and non-visual information about the direction of self-motion contribute to the perception of object motion.

## Discussion

For moving observers to perceive how other objects are moving relative to the stationary environment, the visual system could recover the object-motion component of optic flow. In principle, this could be achieved by factoring out the influence of self-motion using visual self-motion information, non-visual self-motion information, or some combination of both. It is well established that non-visual self-motion information plays a role in recovering the object-motion component of optic flow during walking and head movements [4], [13], [14]. It has also been shown that visual self-motion information influences the perception of object motion during self-motion [6], [7], [18], [19]. In those studies, however, self-motion was simulated rather than real. Therefore, whether or not visual self-motion information plays a role during real, actively generated self-motion remains an open question. In this study, we found that visual self-motion information does play a role even when non-visual self-motion information is also available. However, its contribution was less than would be expected if subjects relied entirely on visual self-motion information. Taken together with previous research [13], we conclude that both visual and non-visual self-motion information contribute to the perception of object motion during self-motion.

The ability to use both visual and non-visual self-motion information may improve the accuracy with which people perceive object motion during self-motion. Although the design of the present study does not allow us to directly test this hypothesis, recent studies on perceptual stability provides indirect support. In one study [20], subjects walked by an object and were instructed to judge whether it was rotating with or against them. Judgments of object rotation were less biased and more precise when the stationary background, which provides self-motion information, was visible. In another study [21], subjects judged whether an object was translating with or against them while they themselves were either actively or passively translating. Judgments of object motion were less biased when the stationary background was visible, even when self-motion was actively generated. These findings indicate that observers’ can better discriminate stationary and moving objects when both visual and non-visual self-motion information are available. Similarly, the availability of visual self-motion information may improve one’s ability to perceive the direction and magnitude of object motion even when non-visual self-motion information is also available.

### The Functional Role of Recovering the Object-motion Component

The ability to recover the object-motion component of optic flow most likely plays an important role in allowing moving observers to perceive the movement of objects relative to the environment (e.g., to perceive whether an object is stationary or moving, its speed, and whether it is on a collision course with another stationary or moving object). What is less clear is whether observers must recover the object-motion component to successfully guide locomotion in the presence of moving objects. On the one hand, it may seem obvious that this process is necessary for tasks such as interception and avoidance of moving objects. Indeed, it is not uncommon for studies on flow parsing to be motivated by statements that reflect such an assumption, as the following quotation from a recent paper on flow parsing demonstrates: “The task of parceling perceived visual motion into self- and object motion components is critical to safe and accurate visually guided navigation” [22]. Similar statements appear in several other studies on flow parsing [3], [19], [23]. On the other hand, many researchers who study interception and obstacle avoidance endorse a strategy (i.e., the bearing angle strategy, described below) that does not require the visual system to recover the object-motion component. In this section, we will attempt to reconcile this discrepancy in the literature.

Arguably the most widely accepted strategy for interception and obstacle avoidance is the bearing angle (BA) strategy. The bearing angle is the direction of the target with respect to a reference direction that remains fixed in exocentric coordinates. By moving so as to keep the target at a fixed bearing angle, the observer will eventually collide with the target. Similarly, moving obstacles can be avoided by moving so as to ensure that the bearing angle does not remain fixed. Numerous studies on humans and other animals (including bats, dragonflies, and fish) suggest that the bearing angle is used to guide interception and obstacle avoidance [24], [25], [26], [27], [28], [29], [30], [31].

The change in the bearing angle reflects the relative motion between the observer and the object. Therefore, this information is available in the combined optic flow field that includes both self-motion and object-motion components, and does not require the visual system to recover the object-motion component of optic flow. However, there are important aspects of interception and obstacle avoidance for which the BA model cannot account [32]. For the purposes of this study, the most significant limitation is that the BA model in its current form ignores the fact that there are limits to how fast one can move. This is a problem because such limits must be taken into account to choose appropriate actions and guide locomotion during both obstacle avoidance and interception. For example, the decision about whether to pass in front of or behind a moving obstacle, which was the task that subjects in the present study performed, must be made in a way that takes into account how fast one is capable of moving. The change in bearing angle specifies whether the observer’s current locomotor speed is sufficient to pass in front or behind. However, because the BA model ignores the fact that there are limits to how fast a person can move, the change in bearing angle does not provide information about whether or not it is within one’s capabilities to pass in front.

**Figure 8:**
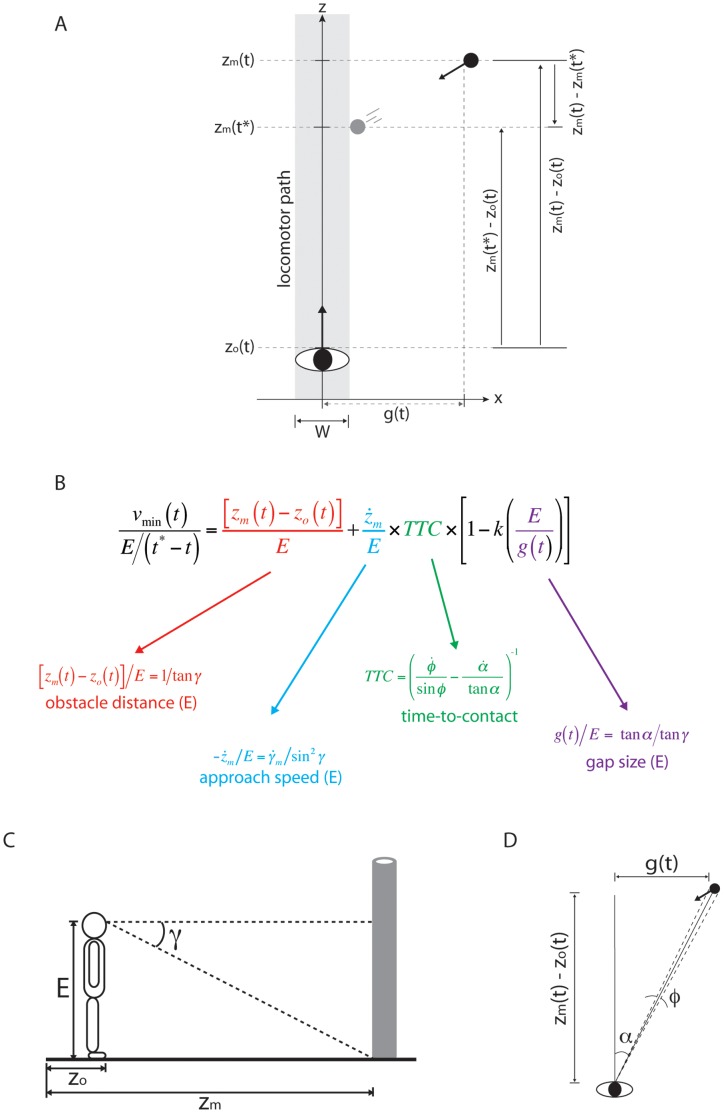
Information about minimum locomotor speed. (A) Top down view of an obstacle crossing an observer’s future path. W is the width of the observer’s body, z_o_ and z_m_ are the positions along the z-axis of the observer and the moving obstacle respectively, g is the spatial gap between the obstacle and the z-axis, and the shaded region is the locomotor path of the observer. (B) Optical specification of Equation 2. (C) Side view of observer and obstacle, showing angular declination of base of obstacle (γ) and eyeheight. (D) Top down view of observer and obstacle, showing visual angle of gap (α) and obstacle (*φ*).

If the change in bearing angle is not useful for perceiving whether to pass in front or behind, then perhaps observers rely on some other source of information. Consider the task used in Experiment 2 of the present study in which subjects judged whether they could safely pass in front of a moving obstacle crossing their future path. In terms of spatial variables, the minimum locomotor speed (*v_min_*) needed to pass in front of the obstacle is equal to the minimum distance that the observer must travel to pass the obstacle divided by the amount of time remaining until the inside edge of the obstacle reaches the locomotor path (gray region in Figure 8A defined by the observer’s body width W):
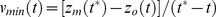
(1)


where *z_m_* and z_o_ are the positions along the z-axis of the moving obstacle and the observer (respectively), and t^*^ is the time at which the inside edge of the obstacle reaches the locomotor path. This is equivalent to:


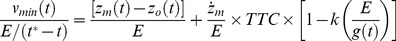
(2)

where *E* is the observer's eyeheight, *ż_m_* is approach speed of the obstacle, *TTC* (time-to-contact) is the amount of time remaining until the obstacle reaches the z-axis, k is a constant equal to *W*/(2 E), and g is the spatial gap between the inside edge of the obstacle and the z-axis. As shown in Figure 8B, each component of Equation 2 is optically specified. That is, there is visual information in the form of optical variables and changes in optical variables for each of the spatial properties in Equation 2. By detecting this information, observers can perceive the minimum locomotor speed needed to safely pass in front of a moving obstacle. (See Appendix S1 for the full derivation Equation 2 and its optical specification.)

Now let us return to the task of perceiving whether it is within one’s capabilities to pass in front of a moving obstacle. The determining factor is the minimum speed needed to pass in front in relation to the observer’s maximum possible speed. Therefore, some critical value of the optically specified *v_min_* that reflects the observer’s maximum possible speed separates situations in which it is within the observer’s capabilities to pass in front from situations in which it is not within the observer’s capabilities to pass in front. Assuming that the observer can learn this critical value (i.e., calibrate the information) through active exploration, he or she can use information about *v_min_* to reliably perceive whether it is within his or her capabilities to pass in front.

In order to detect information about *v_min_*, however, observers must be able to recover the object-motion component of optic flow. This is because the optical specification of 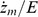 involves 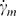, which is the component of 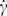 that is due to the motion of the object independent of the observer’s self-motion (see Figure 8B). γ is the visual angle between eye level and the base of the moving object (see Figure 8C). 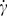 is the rate of change of this angle and is influenced by the movement of both the observer and the object. Specifically, 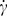 is the sum of 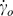 (the rate of change of γ due to the observer’s self-motion) and 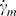 (the rate of change of γ due to object motion). The optical specification of 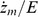 involves 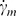. Therefore, detecting information about *v_min_* while moving requires the visual system to factor out the influence of self-motion.

To summarize, although the change in bearing angle is directly available in the combined optic flow field, the BA strategy does not explain how people take their locomotor capabilities into account during interception and obstacle avoidance. The availability of information about *v_min_* (see Figure 8B) offers a possible solution to this problem. However, information about *v_min_* is found in the object-motion component of optic flow. Therefore, the ability to recover the object-motion component of optic flow may play an important role during interception and obstacle avoidance in that it allows observers to detect information that is relevant to successfully guiding locomotion in the presence of moving objects.

Regardless of its role in interception and obstacle avoidance, the ability of moving observers to recover object motion during self-motion plays an important role in perceiving how objects move through the environment. Taken together with previous studies, the findings of the present study suggest that when self-motion is real and actively generated, both visual and non-visual self-motion information are used to factor out the influence of self-motion.

## Supporting Information

Appendix S1 **Derivation of** **Equation 2** **and equations for the optical specification of** ***v_min_*** **.**
(DOCX)
