# Supplementary material for: Visual and Non-Visual Contributions to the Perception of Object Motion during Self-Motion
Source: PLoS One. 2013 Feb 7;8(2):e55446. doi: 10.1371/journal.pone.0055446 (PMC3567075; doi:10.1371/journal.pone.0055446)
Supplement: Appendix S1 — Derivation of Equation 2 and equations for the optical specification of vmin . (DOCX) [file pone.0055446.s001.docx]

# Appendix S1

As illustrated on the right side of Figure 8A, the numerator in Equation 1, $\left[z_{m}\left(t^{*}\right)-z_{o}\left(t\right)\right]$, is equal to the current distance from the observer to the obstacle minus the distance along the z-axis that the obstacle moves between t and t^*^; that is:

$\left[z_{m}\left(t^{*}\right)-z_{o}\left(t\right)\right]=\left[z_{m}\left(t\right)-z_{o}\left(t\right)\right]-\left[z_{m}\left(t\right)-z_{m}\left(t^{*}\right)\right]$ (S1)

The distance along the z-axis that the obstacle moves between t and t^*^ is:

$\left[z_{m}\left(t\right)-z_{m}\left(t^{*}\right)\right]=-\dot{z}_{m}\times TTC\times\left[1-W/\left(2\times g\left(t\right)\right)\right]$ (S2)

$\dot{z}_{m}\times TTC$ is the distance that the obstacle moves from t until the obstacle reaches the z-axis. By multiplying by $\left[1-W/\left(2\times g\left(t\right)\right)\right]$, we get the distance that the obstacle moves from t to t^*^. Substituting Equation S2 into Equation S1 and Equation S1 into Equation 1 yields:

(S3)

Lastly, we substitute 2 × *k* × *E* for W, which allows *g* to be expressed in units of *E* and gives us Equation 2:

(S4)

Next, we show that each component on the right side of Equation 2 (also Equation S4) is optically specified. *z_m_* – *z_o_*, which is the distance along the z-axis from the observer to the obstacle, is optically specified in units of E by:

$\left[z_{m}\left(t\right)-z_{o}\left(t\right)\right]/E=1/\tan\gamma$ (S5)

where γ is the visual angle between eye level and the base of the object (see Figure 8C). $\dot{z}_{m}$ is the speed of the obstacle along the z-axis, which is optically specified in units of E by:

(S6)

(adapted from [[33](#_ENREF_33)]). *TTC*, the amount of time remaining until the obstacle reaches the z-axis, is optically specified by:

(S7)

where is the local visual angle subtended by the inside and outside edges of the obstacle and α is the visual angle between the z-axis and the inside edge of the obstacle [[34](#_ENREF_34)] (see Figure 8D). *g*, which is the size of the spatial gap between the obstacle and the z-axis, is optically specified in units of *E* by:

$g\left(t\right)/E=\tan\alpha/\tan\gamma$ (S8)

[[35](#_ENREF_35),[36](#_ENREF_36)]. Equations S5, S6, S7, and S8 show that each component in Equation 2 is optically specified, and therefore, that *v­_min_* is also optically specified.
